# Efficient fine-tuning of large-scale vision-language models for visual marketing analysis: From brand logo detection to aesthetic preference prediction

**DOI:** 10.1371/journal.pone.0354157

**Published:** 2026-08-07

**Authors:** Suxiang Qin, Songwen Wei

**Affiliations:** Guangxi Normal University for Nationalities, Jiangzhou, Chongzuo, Guangxi, China; Anhui Polytechnic University, CHINA

## Abstract

Visual marketing analysis has emerged as a critical research domain at the intersection of computer vision, natural language processing, and consumer behavior modeling. This study addresses three fundamental challenges in this field: the accurate and robust detection of brand logos in complex commercial visual scenes, the construction of a unified model for understanding both visual content and accompanying text through fine-grained vision-language alignment, and the quantification of audience aesthetic preferences for data-driven marketing effectiveness prediction. We propose Brand-Aesthetic Vision-Language Assistant (BAVLA), a novel framework comprising Multi-granularity Context-aware Brand Detection and Fusion Module (MCBF), Aesthetic-aware Vision-Language Alignment and Reasoning Module (AVLR), and Task-aware Progressive Efficient Tuning strategy (TaPET). Compared to existing methods, the proposed MCBF module improves logo detection mAP by 6.2% and 2.5% over Faster R-CNN and YOLOv8, respectively, on the Flickr Logo-27 dataset. Furthermore, the complete BAVLA framework achieves superior aesthetic prediction performance, surpassing previous best methods (NIMA, AestheticCNN) by 0.084 and 0.055 in PLCC, respectively, on the AVA dataset, while attaining 82.4% accuracy in marketing effectiveness classification. These findings validate the effectiveness of the proposed modules and training strategy in advancing visual marketing analysis capabilities.

## Introduction

Visual marketing analysis represents a rapidly evolving research domain that bridges computer vision, natural language processing, and consumer psychology, holding substantial commercial significance in the contemporary digital economy [1]. The proliferation of visual content across social media platforms, e-commerce websites, and digital advertising channels has generated an unprecedented volume of marketing images that contain rich semantic information about brand identities, product presentations, and consumer engagement patterns [2]. Companies increasingly rely on automated systems to analyze these visual materials for brand monitoring, advertising optimization, and consumer response prediction. The ability to automatically detect brand logos within complex visual scenes, understand the alignment between visual elements and accompanying textual descriptions, and quantify the aesthetic appeal that influences consumer preferences has become essential for data-driven marketing strategies [3]. This technological capability enables businesses to monitor brand exposure, evaluate advertising effectiveness, and personalize content recommendations at scales that would be impossible through manual analysis.

Recent advances in deep learning have produced significant progress across multiple research areas relevant to visual marketing analysis [4]. Object detection methodologies have evolved from traditional region-based approaches to modern anchor-free and transformer-based frameworks [5], achieving remarkable improvements in detection accuracy and computational efficiency. Concurrently, vision-language pretraining has established new paradigms for multi-modal understanding, with large-scale models demonstrating emergent capabilities in cross-modal reasoning and zero-shot transfer [6]. Aesthetic assessment research has developed computational frameworks for evaluating visual appeal based on composition, color harmony, and artistic principles [7]. However, despite these individual advancements, existing methods face substantial challenges when applied to the integrated task of visual marketing analysis. Brand logo detection in real-world marketing images encounters difficulties with occlusion, viewpoint variation, and context interference from surrounding visual elements [8]. Vision-language understanding in marketing contexts requires not only surface-level alignment but also deep semantic reasoning about brand-message consistency. Aesthetic preference prediction demands subjective judgments that vary across demographic groups and cultural contexts [9].

Three fundamental problems persist as major obstacles in visual marketing analysis research. The first challenge involves efficiently extracting and identifying diverse brand logos from complex, variable commercial visual scenes such as advertisements, posters, and social media images with high precision and robustness [3]. Traditional object detection methods often struggle with the unique characteristics of brand logos, including their extreme aspect ratios, partial occlusions in composite marketing layouts, and the presence of stylized or modified visual representations that deviate from canonical logo designs. The second challenge transcends simple object detection to construct a unified model capable of simultaneously understanding visual content encompassing objects, scenes, and brand elements alongside accompanying textual content including slogans, descriptions, and marketing copy, thereby enabling fine-grained vision-language alignment and reasoning about semantic consistency [10]. Current vision-language models pretrained on general web data may not adequately capture the domain-specific relationships between brand visual elements and marketing textual messages that characterize effective advertising. The third challenge requires developing quantitative frameworks for measuring and predicting the audience aesthetic responses evoked by marketing images, providing interpretable, data-driven metrics for marketing effectiveness prediction that can guide content optimization and campaign evaluation.

This research proposes BAVLA, a comprehensive framework designed to address the aforementioned challenges through three interconnected components operating in a cascaded pipeline. The first component, denoted as Multi-granularity Context-aware Brand Detection and Fusion Module (MCBF), employs a lightweight visual backbone network to extract multi-scale visual features and introduces a novel context attention mechanism that dynamically aggregates contextual information from surrounding elements such as products, human figures, and background scenes. This module incorporates deformable convolution networks to enhance detection capabilities for irregular, occluded, or small-scale logos. The second component, termed Aesthetic-aware Vision-Language Alignment and Reasoning Module (AVLR), constructs a lightweight multi-modal transformer encoder that integrates the context-enhanced brand visual features from MCBF with paired textual descriptions. A distinguishing feature of AVLR is its aesthetic-aware cross-modal attention mechanism that introduces learnable aesthetic prior vectors to guide the model toward visual elements contributing to aesthetic judgments such as composition, color coordination, and brand prominence. The third component, named Task-aware Progressive Efficient Tuning strategy (TaPET), provides a systematic approach for efficient fine-tuning of large-scale pretrained vision-language models on downstream visual marketing tasks while preserving pretrained knowledge and avoiding catastrophic forgetting.

The contributions of this research are threefold.

- First, we propose MCBF module that introduces an innovative context attention and deformable convolution fusion mechanism specifically designed for brand logo detection in complex commercial scenes, achieving superior performance compared to conventional object detection approaches that treat logos as generic objects without considering their contextual relationships. Specifically, its novelty lies in the dynamic context aggregation from surrounding marketing scene elements and adaptive receptive fields via deformable convolutions, which are jointly optimized for robust logo localization amidst occlusions and stylistic variations.
- Second, AVLR module represents a novel architecture that integrates aesthetic prior knowledge into cross-modal attention mechanisms, enabling the model to perceive and predict image aesthetic preferences while performing vision-language alignment, thereby bridging the gap between recognition and aesthetic evaluation in marketing contexts. Specifically, its core innovation is the learnable aesthetic bias matrix that modulates cross-modal attention scores, guiding the model to focus on visual-linguistic associations most relevant to aesthetic judgment, a capability absent in general-purpose vision-language models.
- Third, the TaPET strategy provides a task-aware progressive fine-tuning framework that systematically activates and optimizes different components of the model based on task relationships, combined with parameter-efficient tuning techniques to enable stable and efficient adaptation of large-scale vision-language models to data-scarce visual marketing domains. Specifically, its novelty stems from the curriculum that progresses from general alignment to brand-specific and then aesthetic-focused tuning, which prevents catastrophic forgetting and interference, ensuring efficient knowledge transfer with minimal trainable parameters.

The key advantages of BAVLA in addressing the three identified problems include its unified architecture that handles detection, understanding, and prediction tasks without requiring task-specific model ensembles, its efficient fine-tuning strategy that enables deployment with limited training data while leveraging knowledge from large-scale pretrained models, and its interpretable aesthetic attention mechanisms that provide insights into the visual factors driving marketing effectiveness predictions. Furthermore, the modular design allows independent optimization of each component and facilitates potential extensions to additional marketing analysis tasks such as sentiment analysis, engagement prediction, and campaign performance attribution.

The remainder of this paper is organized as follows. Section 2 reviews related work on brand logo detection, vision-language learning, aesthetic assessment, and efficient fine-tuning methods. Section 3 presents the detailed methodology of BAVLA, including the MCBF module, AVLR module, and TaPET strategy. Section 4 describes the experimental setup, datasets, comparison studies, ablation studies, and discussion of results. Section 5 provides conclusions and outlines future research directions.

## Related work

### Brand logo detection and recognition

Brand logo detection has attracted significant research attention due to its practical applications in brand monitoring, trademark enforcement, and advertising analysis [8]. Early approaches to logo detection primarily relied on hand-crafted visual features such as SIFT [11], SURF [12], and HOG descriptors [13] combined with bag-of-words representations and support vector machine classifiers [14]. These methods demonstrated moderate performance on controlled datasets but suffered from substantial degradation when applied to real-world images with challenging viewing conditions. The advent of deep learning revolutionized logo detection through the adoption of convolutional neural network architectures that learned hierarchical feature representations directly from image data [15]. Region-based convolutional neural networks including Faster R-CNN [16] and its variants established strong baselines for logo detection by combining region proposal networks with classification and bounding box regression heads. These approaches achieved significant improvements in detection accuracy but incurred substantial computational costs that limited their applicability to real-time or large-scale applications.

Subsequent research explored more efficient detection architectures inspired by single-stage detectors such as YOLO [17] and SSD [18] that eliminated the separate region proposal stage to achieve faster inference speeds. Methods including Tiny-YOLO [19] and MobileNet-based detectors [20] demonstrated the feasibility of deploying logo detection on resource-constrained devices while maintaining reasonable accuracy levels. However, these general-purpose object detectors often struggled with the unique characteristics of brand logos including their extreme aspect ratios, repetitive textual elements, and frequent partial occlusions within composite marketing layouts. Recent work has attempted to address these challenges through specialized architectures incorporating attention mechanisms [21] for focusing on discriminative logo regions and context aggregation modules for capturing surrounding visual information. Despite these advances, existing logo detection methods typically treat logos as isolated objects without explicitly modeling the semantic relationships between logos and other visual elements in marketing compositions, which limits their effectiveness for comprehensive visual marketing analysis tasks that require understanding how logos interact with their visual context.

### Vision-language pretraining and multi-modal understanding

Vision-language pretraining has emerged as a powerful paradigm for learning unified representations that bridge visual and textual modalities, enabling a wide range of downstream applications including image captioning, visual question answering, and cross-modal retrieval [22]. Foundation models such as CLIP [23] and ALIGN have demonstrated remarkable zero-shot and few-shot transfer capabilities by training on massive collections of image-text pairs collected from the web. These models learn to align visual and textual embeddings through contrastive learning objectives that encourage matching image-text pairs to have similar representations while pushing non-matching pairs apart. The success of these approaches has motivated extensive research into improving vision-language representations through architectural innovations, training objectives, and data curation strategies.

Recent advances in vision-language modeling have explored more sophisticated attention mechanisms for cross-modal interaction [24], moving beyond simple late fusion approaches to enable deeper integration of visual and linguistic information. Methods such as BLIP, Flamingo, and LLaVA [25] have demonstrated that large language models can be effectively adapted for visual input through learned projection layers and instruction tuning, enabling conversational interactions about visual content. However, these general-purpose vision-language models pretrained on web-scale data may not adequately capture the domain-specific relationships between visual marketing elements and textual brand messaging that characterize effective advertising. The alignment between brand logos and marketing text involves nuanced semantic connections that go beyond simple object-label correspondences to encompass brand positioning, value propositions, and emotional appeals. Furthermore, existing vision-language models lack explicit mechanisms for incorporating aesthetic judgments that are central to marketing effectiveness evaluation [26].

### Efficient fine-tuning for vision-language models

The exponential growth in scale and capability of pretrained vision-language models has created significant challenges for adapting these models to downstream applications, particularly in domains with limited training data. Full fine-tuning of large-scale models requires updating billions of parameters, which demands substantial computational resources and extensive training data while risking catastrophic forgetting of the valuable knowledge encoded in pretrained representations [27]. These challenges have motivated extensive research into parameter-efficient fine-tuning methods that enable effective adaptation while minimizing the number of trainable parameters and computational costs [28]. Prominent approaches include adapter methods [29] that insert small trainable modules between layers of pretrained models, prefix tuning [30] that prepends learnable continuous prompts to model inputs, and low-rank adaptation (LoRA) that represents weight updates as low-rank factorized perturbations.

Adapter-based methods insert bottleneck layers containing trainable down-projection and up-projection matrices into each transformer block, with the pretrained model weights remaining frozen during training. [31]. This approach has demonstrated competitive performance across various tasks while reducing trainable parameters by orders of magnitude compared to full fine-tuning. Prefix tuning prepends a sequence of learnable vectors to the key and value matrices of attention mechanisms, enabling the model to condition its behavior on task-specific prompts without modifying pretrained weights. Low-rank adaptation factorizes weight updates into low-rank matrices that are added to the original weights during inference, achieving parameter efficiency through the low-rank constraint while maintaining the representational capacity of pretrained models [32]. These methods have proven particularly valuable for adapting large language models and vision-language models to specialized domains where training data is limited or expensive to obtain.

Despite the success of parameter-efficient fine-tuning techniques, applying these methods to complex multi-task scenarios such as visual marketing analysis requires careful consideration of task relationships and training dynamics. Naive joint fine-tuning of multiple tasks may lead to interference between task-specific updates, while sequential fine-tuning may suffer from knowledge forgetting [33]. Our proposed TaPET strategy addresses these challenges by analyzing the relationships among downstream visual marketing tasks and designing a progressive training curriculum that activates different components of the model in an ordered manner, ensuring stable convergence and effective knowledge transfer across tasks.

## Methodology

### Overview

BAVLA employs a three-stage pipeline architecture designed to address the comprehensive requirements of visual marketing analysis, encompassing brand logo detection, cross-modal understanding, and aesthetic preference prediction. The model takes as input a marketing image along with associated textual content such as slogans, descriptions, or captions, and produces structured outputs including detected brand logos with their positions, cross-modal consistency assessments, and predicted aesthetic preference scores. The processing begins with a lightweight visual backbone network that extracts multi-scale feature representations from the input image, which are then processed by the MCBF module to generate context-enhanced brand visual features. These features, encoding both the discriminative characteristics of brand logos and their relationships with surrounding visual elements, are subsequently fed to the AVLR module along with the input text for joint multi-modal encoding. The AVLR module produces unified cross-modal representations that capture semantic correspondences between visual and textual content while encoding aesthetic-aware attention patterns. Finally, the TaPET strategy governs the training process, prescribing the sequence and methodology for updating the parameters of MCBF, AVLR, and the pretrained vision-language backbone to achieve optimal adaptation to the downstream visual marketing tasks while maintaining computational efficiency.

The training process of BAVLA comprises three progressive stages, enabling the model to gradually adapt to more specialized tasks. The first stage employs large-scale image-text matching data for general visual-language alignment training, allowing the model’s underlying architecture to adapt to fundamental correspondences between visual and textual features. The second stage focuses on training with brand-related data, activating the context attention mechanism and deformable convolutional layers within the MCBF module while fine-tuning the cross-modal attention head in the AVLR module. The third stage further optimizes the aesthetic perception attention mechanism and correlation prediction head within the AVLR module using aesthetic preference data, achieving specialization for aesthetic evaluation. Throughout the training process, parameter-efficient fine-tuning techniques like LoRA and Adapter are employed, enabling efficient adaptation to large-scale pre-trained models by updating only a small subset of parameters.

**Fig 1:**
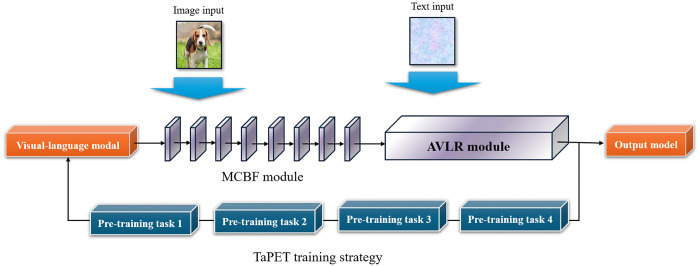
The architecture diagram of the proposed model.

Fig 1 presents the architecture diagram of the proposed model. The following subsections provide detailed descriptions of each component.

### Multi-granularity context-aware brand detection and fusion module

**Fig 2:**
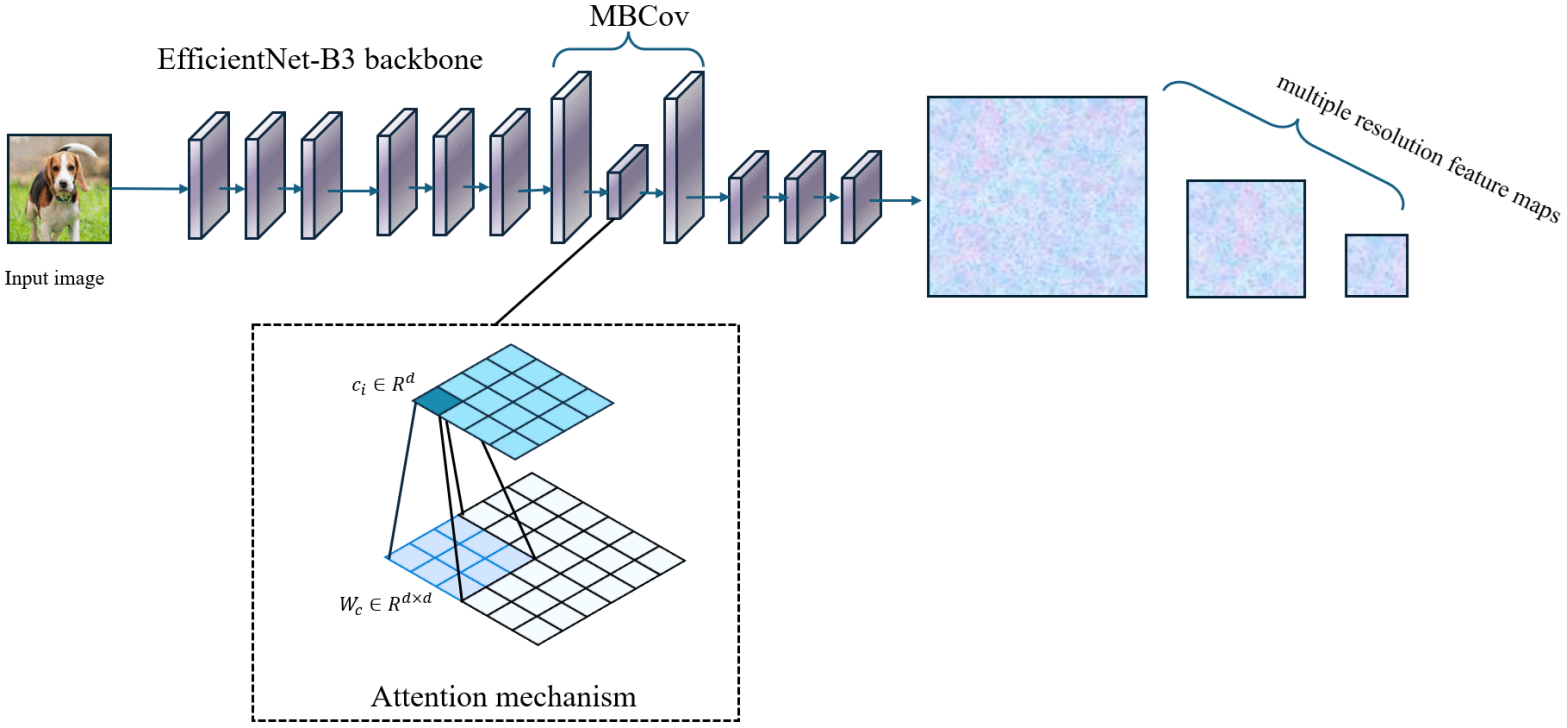
The structure of the MCBF module.

As shown in Fig 2, the MCBF module addresses the challenge of precise and robust brand logo detection in complex commercial visual scenes through a multi-stage architecture that combines multi-scale feature extraction, context attention aggregation, and deformable convolution enhancement. Given an input image $I\in {\mathbb{R}}^{H\times W\times 3}$, the module first extracts multi-scale visual features using a lightweight backbone network *E*_vis_ based on EfficientNet-B3 architecture, producing feature maps at multiple resolution levels $F_{l}=E_{\text{vis}}^{l}(I)$ for l∈{1,2,3,4} with spatial dimensions $H/2^{l}\times W/2^{l}$.

To enhance the feature representations for logo detection, MCBF introduces a context attention mechanism that dynamically aggregates contextual information from surrounding visual regions. The attention computation can be formulated as follows. Given a query feature vector $q\in {\mathbb{R}}^{d}$ extracted from a candidate logo region and context feature vectors $c_{i}\in {\mathbb{R}}^{d}$ from surrounding regions, the context attention weight for each context region is computed as:


$$\begin{array}{c} \alpha_{i}=\frac{\exp(\text{score}(q,c_{i}))}{\sum_{j=1}^{N}\exp(\text{score}(q,c_{j}))},\;\text{where}\;\text{score}(q,c)=q^{⊤}W_{c}c, \end{array}$$ (1)

where $W_{c}\in {\mathbb{R}}^{d\times d}$ is a learnable projection matrix and *N* denotes the number of context regions considered. The context-enhanced feature is then obtained through weighted aggregation:


$$\begin{array}{c} \tilde{q}=q+\sum_{i=1}^{N}\alpha_{i}(W_{v}c_{i}), \end{array}$$ (2)

where $W_{v}\in {\mathbb{R}}^{d\times d}$ is a value projection matrix. This attention mechanism enables the model to dynamically adjust the influence of different contextual elements based on their relevance to the target logo region.

To handle irregular logo shapes, partial occlusions, and scale variations commonly encountered in marketing images, MCBF incorporates deformable convolution layers that learn adaptive sampling locations for each convolution operation. The deformable convolution with *K* sampling offsets can be expressed as:


$$\begin{array}{c} y(p)=\sum_{k=1}^{K}w_{k}\cdot x(p+p_{k}+\Delta p_{k})\cdot \Delta m_{k}, \end{array}$$ (3)

where *p* denotes the current spatial location, $w_{k}$ are the convolution weights, $p_{k}$ are the predefined sampling offsets, $\Delta p_{k}$ are the learnable offset displacements, and $\Delta m_{k}\in [0,1]$ are the learnable modulation scalars controlling the contribution of each sampling location. The offsets and modulations are predicted by separate convolutional layers operating on the input features, enabling the model to learn task-specific receptive field adaptations.

The MCBF module outputs a sequence of brand visual feature vectors ${𝐯}_{\text{brand}}=[v_{1},v_{2},...,v_{M}]$ where each vector encodes both the discriminative appearance of a detected logo instance and its enriched contextual information, along with preliminary brand category and position estimates $B=\{(c_{k},b_{k}){\}}_{k=1}^{M}$ where $c_{k}$ represents the predicted brand category and $b_{k}=(x_{k},y_{k},w_{k},h_{k})$ denotes the bounding box coordinates. These outputs serve as the foundation for subsequent visual-language alignment in the AVLR module.

The integration of context attention with deformable convolution provides MCBF with complementary capabilities for handling the diverse challenges of brand logo detection. The context attention mechanism enables the model to leverage surrounding visual elements such as products, human figures, and background scenes for improved logo recognition, particularly beneficial for cases where logos appear in modified or stylized forms. The deformable convolution provides flexible receptive fields that can adapt to irregular logo shapes and partially occluded instances, enhancing detection robustness in complex marketing compositions.

### Aesthetic-aware vision-language alignment and reasoning module

**Fig 3:**
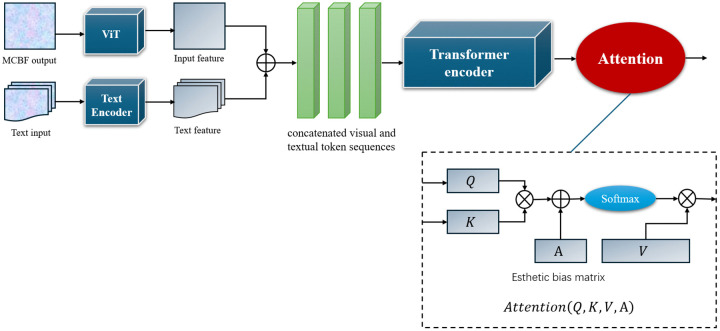
The structure of the AVLR module.

As shown in Fig 3, the AVLR module constructs a unified multi-modal representation that jointly processes the context-enhanced brand visual features from MCBF and the paired textual descriptions, enabling fine-grained cross-modal alignment and aesthetic preference prediction. The module employs a lightweight multi-modal transformer encoder *T*_mm_ that processes the concatenated visual and textual token sequences. Visual tokens consist of the brand feature vectors ${𝐯}_{\text{brand}}$ along with additional regional visual features extracted from the input image through a vision transformer encoder. Textual tokens are obtained by applying a pretrained text encoder to the marketing text inputs and extracting the last hidden states.

The distinguishing characteristic of AVLR is its aesthetic-aware cross-modal attention mechanism that incorporates learnable aesthetic prior vectors to guide the attention computation toward visual-linguistic associations that contribute to aesthetic perception. The aesthetic-aware attention computation extends the standard cross-attention mechanism as follows:


$$\begin{array}{c} \text{Attention}(Q,K,V,𝒜)=\text{softmax}\left(\frac{QK^{⊤}}{\sqrt{d_{k}}}+𝒜\right)V, \end{array}$$ (4)

where *Q*, *K*, and *V* represent the query, key, and value matrices from the standard attention formulation, and $𝒜\in {\mathbb{R}}^{L_{v}\times L_{t}}$ is a learned aesthetic bias matrix that modulates the attention scores based on aesthetic relevance, with $L_{v}$ and $L_{t}$ denoting the numbers of visual and textual tokens respectively.

The aesthetic bias matrix 𝒜 is generated through a shallow neural network that takes as input the concatenation of visual and textual features, producing a relevance-weighted attention modulation. This mechanism can be expressed as:


$$\begin{array}{c} 𝒜=W_{2}(\text{ReLU}(W_{1}([\bar{v},\bar{t}])))+b, \end{array}$$ (5)

where $\bar{v}\in {\mathbb{R}}^{d}$ and $\bar{t}\in {\mathbb{R}}^{d}$ represent pooled visual and textual features, $W_{1}\in {\mathbb{R}}^{2d\times d_{a}}$ and $W_{2}\in {\mathbb{R}}^{d_{a}\times (L_{v}\times L_{t})}$ are learnable weight matrices, and $d_{a}$ is the dimensionality of the intermediate aesthetic feature space.

AVLR employs a multi-task learning objective that combines cross-modal contrastive learning, masked language modeling, and task-specific predictions. The cross-modal contrastive loss enforces alignment between matched visual-text pairs while pushing apart mismatched combinations:


$$\begin{array}{c} {ℒ}_{\text{cl}}=-\log\frac{\exp(\text{sim}(v_{i},t_{i})/\tau )}{\sum_{j=1}^{N}\exp(\text{sim}(v_{i},t_{j})/\tau )}, \end{array}$$ (6)

where sim(·,·) denotes the cosine similarity between visual and textual representations, and τ is a learnable temperature parameter. The masked language modeling loss reconstructs masked tokens based on cross-modal context:


$$\begin{array}{c} {ℒ}_{\text{mlm}}={𝔼}_{\left(v,t\right)}\left[-\sum_{m\in M}\log p(t_{m}|v,t⧵m)\right], \end{array}$$ (7)

where *M* is the set of masked token positions. The task-specific prediction heads produce outputs for brand detection consistency, information alignment, and aesthetic preference score, with corresponding regression or classification losses.

The unified cross-modal representation produced by AVLR captures both the semantic correspondences between visual brand elements and textual marketing content and the aesthetic characteristics that influence viewer preferences. The aesthetic-aware attention mechanism provides interpretability by revealing which visual-linguistic associations contribute most strongly to aesthetic judgments, enabling analysis of the visual factors driving marketing effectiveness.

The outputs of AVLR include a unified cross-modal representation ${𝐡}_{\text{mm}}$ that integrates visual brand information, textual content, and aesthetic-aware features, along with task-specific predictions ${\hat{y}}_{\text{brand}}$ for logo detection results, ${\hat{y}}_{\text{align}}$ for vision-language consistency scores, and ${\hat{y}}_{\text{aesthetic}}$ for aesthetic preference ratings. These predictions collectively enable comprehensive visual marketing analysis spanning brand exposure monitoring, content-audience alignment evaluation, and marketing effectiveness prediction.

### Task-aware progressive efficient tuning strategy

The TaPET strategy provides a systematic framework for efficient fine-tuning of large-scale pretrained vision-language models on the downstream visual marketing analysis tasks while preserving pretrained knowledge and achieving stable convergence. The key insight underlying TaPET is that the four downstream tasks including brand detection, text-image matching, aesthetic score prediction, and marketing effectiveness classification exhibit varying degrees of relatedness that can be exploited through a progressive training curriculum.

TaPET first analyzes the relationships among the downstream tasks through a task affinity computation that measures the similarity of task gradients with respect to the pretrained model parameters. Given the pretrained model parameters $\theta_{\text{pretrain}}$, the task affinity between tasks *a* and *b* is computed based on the cosine similarity of their gradient vectors:


$$\begin{array}{c} \text{Affinity}(a,b)=\frac{\sum_{i\in \theta_{\text{pretrain}}}g_{i}^{a}\cdot g_{i}^{b}}{\left\|g^{a}\|\cdot \|b^{b}\right\|}, \end{array}$$ (8)

where $g^{a}$ and $g^{b}$ represent the gradient vectors for tasks *a* and *b* computed on samples from their respective training distributions. Tasks with higher affinity values share more similar gradient directions, indicating that joint optimization is likely to be beneficial.

**Fig 4:**
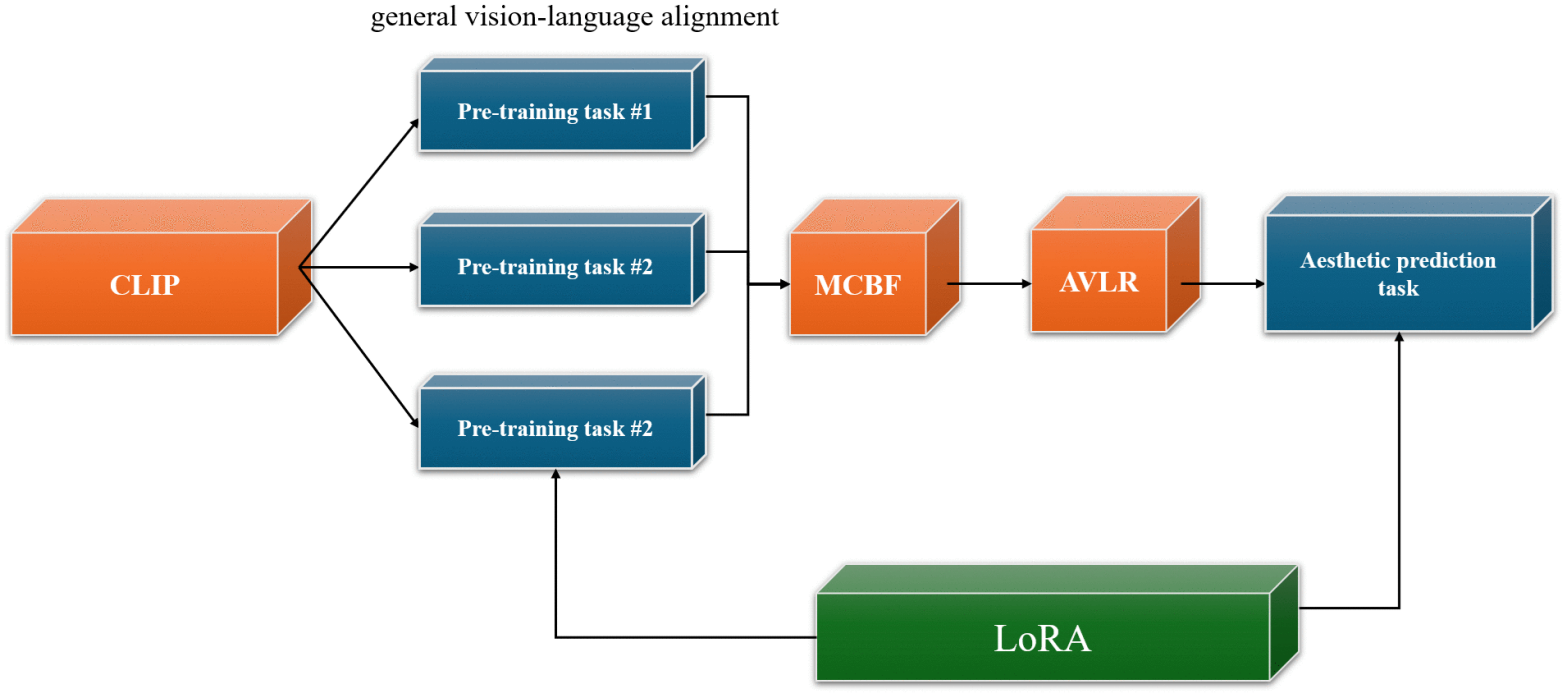
The principle of the TaPET strategy.

As shown in Fig 4, based on the task affinity analysis, TaPET designs a progressive training curriculum consisting of three phases. Phase 1 performs general vision-language alignment using task *a* with highest pretraining similarity, such as image-text matching, to gently adapt the lower layers of the pretrained model. During this phase, only the adapter modules inserted in the lower transformer layers and the projection layers of the visual and text encoders are trained, with the core attention and feed-forward parameters remaining frozen. Phase 2 activates the MCBF module and the cross-modal attention heads in AVLR using brand-related data for task *b*, optimizing the parameters that capture brand-specific visual patterns and their textual correspondences. The adapter modules in middle transformer layers are unfrozen to enable deeper representations of brand information. Phase 3 specializes in aesthetic prediction using task *c* with strongest aesthetic focus, primarily updating the aesthetic-aware attention mechanisms and prediction heads while maintaining the previously learned representations.

Throughout the training process, TaPET employs parameter-efficient fine-tuning techniques including LoRA for attention weight updates and adapter modules for feed-forward transformations. The LoRA update can be expressed as:


$$\begin{array}{c} W_{\text{new}}=W_{\text{pretrain}}+\Delta W,\;\Delta W=A\cdot B,\;A\in {\mathbb{R}}^{d\times r},B\in {\mathbb{R}}^{r\times k}, \end{array}$$ (9)

where *W*_pretrain_ is the frozen pretrained weight matrix, ΔW is the low-rank update with rank r≪min(d,k), and *A* and *B* are trainable low-rank factor matrices. This parameterization reduces the number of trainable parameters by a factor of approximately (*d* + *k*)/*r* compared to full fine-tuning.

The progressive nature of TaPET ensures that the model’s foundational capabilities for visual-language understanding are established before task-specific specializations are introduced. This curriculum prevents the instability that can arise from attempting to learn multiple diverse objectives simultaneously from random initialization, while the task-aware ordering leverages the transferability of related skills to improve sample efficiency on later tasks. The combination of progressive activation with parameter-efficient updates enables BAVLA to achieve strong performance on the visual marketing analysis tasks despite the limited availability of domain-specific training data.

The TaPET strategy directly influences the training dynamics of MCBF and AVLR by prescribing which parameters to update at each training phase and which to maintain frozen. This systematic approach ensures that the visual detection capabilities of MCBF are consolidated in Phase 2 before the complex cross-modal reasoning of AVLR is introduced in Phase 3, avoiding the catastrophic forgetting or gradient interference that could occur with naive joint training. The result is a stable and efficient fine-tuning process that produces a well-adapted BAVLA model capable of comprehensive visual marketing analysis.

## Experiment

### Experimental setup

The experiments were conducted on a computing cluster equipped with NVIDIA A100 GPUs (80GB memory) and AMD EPYC 7763 processors. The training and evaluation framework was implemented in PyTorch 2.0 with the Transformers and Detectron2 libraries. For the visual backbone, we employed EfficientNet-B3 as the MCBF feature extractor, initialized with ImageNet pretrained weights. The multi-modal transformer encoder used in AVLR consisted of 6 transformer layers with 8 attention heads per layer and a hidden dimension of 512, built upon the CLIP ViT-L/14 architecture. The parameter-efficient tuning employed LoRA with rank 16 for attention weight updates and adapter modules with bottleneck dimension 64 inserted after each transformer layer. The total number of trainable parameters was approximately 45 million, representing less than 3% of the full CLIP model parameters. The optimal parameters and configurations of the proposed BAVLA model is shown in Table 1.

**Table 1:** Optimal parameters and configurations of the proposed BAVLA model.

Component/Parameter	Optimal Configuration
**Model Architecture**	
Visual Backbone for MCBF	EfficientNet-B3 (ImageNet pretrained)
Multi-modal Encoder Layers (AVLR)	6
Attention Heads per Layer	8
Hidden Dimension	512
Base Vision-Language Model	CLIP ViT-L/14
**Training Hyperparameters**	
Optimizer	AdamW
Weight Decay	0.01
Batch Size	64
Phase 1: General Alignment LR	$1\times 10^{-4}$ (cosine decay to $1\times 10^{-5}$)
Phase 2: Brand-specific LR	$5\times 10^{-5}$
Phase 3: Aesthetic-focused LR	$2\times 10^{-5}$
Image Size	448×448
**Parameter-Efficient Tuning (TaPET)**	
LoRA Rank (*r*)	16
Adapter Bottleneck Dimension	64
Total Trainable Parameters	~45 Million (<3% of full CLIP)

The training process followed the three-phase TaPET curriculum with the following configurations. Phase 1 used a learning rate of 1e-4 with linear warmup over 500 iterations and cosine decay to 1e-5, trained for 10 epochs on the general image-text matching data. Phase 2 reduced the learning rate to 5e-5 for fine-tuning the MCBF module and AVLR cross-modal attention heads, with a training duration of 15 epochs on brand detection data. Phase 3 employed a learning rate of 2e-5 for optimizing the aesthetic-aware attention mechanisms and prediction heads, trained for 20 epochs on aesthetic scoring data. All training phases used the AdamW optimizer with weight decay 0.01 and batch size 64 distributed across 4 GPUs. The input images were resized to 448×448 pixels with random horizontal flips and color jitter augmentations applied during training.

The Low-Rank Adaptation (LoRA) parameters were initialized following the standard practice to ensure training stability and a zero start. For each low-rank pair ΔW=BA, where $A\in {\mathbb{R}}^{r\times d}$ and $B\in {\mathbb{R}}^{d\times r}$ with rank *r* = 16, matrix 𝐀 was initialized using Kaiming uniform initialization, and matrix 𝐁 was initialized to zeros. This initialization guarantees that the low-rank update ΔW is zero at the beginning of training, so the fine-tuning process starts from the exact pre-trained weights without causing an initial perturbation. The LoRA modules were applied to the query and value projection matrices within all self-attention and cross-attention layers of the vision-language encoder. A constant scaling factor of α/r=32/16=2 was used to merge the low-rank update with the frozen pre-trained weights during inference. A dropout rate of 0.1 was applied to the LoRA activations for regularization. This identical LoRA configuration and initialization scheme was maintained consistently across all three progressive training phases (General Alignment, Brand-specific, and Aesthetic-focused) of the TaPET strategy to ensure stable adaptation and cumulative learning.

The evaluation employed multiple metrics appropriate to each task. For brand detection, we used Average Precision (AP) computed as the area under the precision-recall curve at IoU threshold 0.5, and mean AP (mAP) averaged across all brand categories:


$$\begin{array}{c} \text{AP}=\int_{0}^{1}P(r)\;dr,\;\text{mAP}=\frac{1}{C}\sum_{c=1}^{C}{\text{AP}}_{c}, \end{array}$$ (10)

where *P*(*r*) denotes precision at recall level *r* and *C* represents the number of brand categories. For classification tasks including vision-language consistency and marketing effectiveness prediction, we reported accuracy, precision, recall, and F1-score. For aesthetic score regression, we used Pearson Linear Correlation Coefficient (PLCC) measuring linear correlation, Spearman Rank Correlation Coefficient (SRCC) measuring monotonic relationship, and Root Mean Square Error (RMSE) quantifying prediction error:


$$\begin{array}{c} \text{PLCC}=\frac{\sum_{i=1}^{n}(y_{i}-\bar{y})({\hat{y}}_{i}-\bar{\hat{y}})}{\sqrt{\sum_{i=1}^{n}(y_{i}-\bar{y}{)}^{2}}\sqrt{\sum_{i=1}^{n}({\hat{y}}_{i}-\bar{\hat{y}}{)}^{2}}},\;\text{SRCC}=1-\frac{6\sum_{i=1}^{n}d_{i}^{2}}{n(n^{2}-1)}, \end{array}$$ (11)

where $y_{i}$ and ${\hat{y}}_{i}$ are the ground truth and predicted scores for sample *i*, $\bar{y}$ and $\bar{\hat{y}}$ are their means, and $d_{i}$ is the rank difference between $y_{i}$ and ${\hat{y}}_{i}$. For advertising optimization evaluation, we employed Normalized Discounted Cumulative Gain (nDCG) measuring the ranking quality of candidate image versions.

### Model generalization

The generalization capability of the BAVLA framework stems from several key design and training strategies. First, the model is built upon the CLIP (ViT-L/14) foundation, which is pre-trained on a massive and diverse dataset of image-text pairs collected from the web. This provides the model with a robust and generalized understanding of visual concepts and their associations with language, serving as a strong prior for downstream tasks. Second, the proposed Task-aware Progressive Efficient Tuning (TaPET) strategy is explicitly designed to preserve this generalized knowledge. By freezing the majority of the pre-trained parameters and only selectively updating a small subset via parameter-efficient techniques (LoRA and Adapters), the risk of catastrophic forgetting is minimized. The progressive curriculum—starting from general vision-language alignment, then specializing in brand-related features, and finally focusing on aesthetic prediction—ensures that task-specific adaptations are built incrementally on top of stable, generalized representations, rather than overwriting them. Third, standard data augmentation techniques (random horizontal flips, color jitter) applied during training introduce variability and improve robustness to common image transformations. While the cross-dataset experiments noted in the Conclusion indicate that performance can degrade when transferring between distinct marketing domains (e.g., from social media ads to product packaging), the aforementioned strategies collectively enhance the model’s ability to generalize within related visual marketing contexts and provide a principled base for further domain adaptation if required.

### Datasets

As shown in Table 2, the experiments utilized two benchmark datasets selected for their relevance to the core tasks of brand logo detection and aesthetic preference prediction.

**Table 2:** Comparison of fundamental properties between two datasets.

Characteristic	Flickr Logo-27	AVA Aesthetic Analysis
Total Images	6,812 images	250,000 images
Image Source	Flickr platform	DPChallenge website
Brand Categories	27 distinct logo classes	Not applicable (aesthetic only)
Annotation Type	Bounding box annotations	Binary aesthetic scores (mean > 5: aesthetic)
Image Resolution	Various resolutions, avg 500×500	500×500 pixels
Average Logos per Image	1.2 logos	Not applicable
Image Quality	Real-world photos with varied conditions	User-submitted photographs
Label Distribution	Imbalanced (8–654 images per class)	Balanced distribution (approx. 50−50 split)
Typical Use Cases	Logo detection, brand recognition	Aesthetic quality prediction

Flickr Logo-27 dataset [34] provides a standard benchmark for logo detection and recognition, containing 6,812 images harvested from the Flickr platform featuring 27 distinct brand logos including prominent commercial brands such as Adidas, Apple, BMW, Coca-Cola, Ferrari, McDonald’s, Nike, Starbucks, and others. Each image contains bounding box annotations indicating the positions of visible logos, with an average of 1.2 logos per image and substantial variation in logo size, orientation, occlusion level, and contextual background. The dataset exhibits significant class imbalance with some categories containing as few as 8 images while others have over 600 examples, presenting challenges for balanced learning across brand categories. The real-world nature of the images, captured under diverse lighting conditions, viewpoints, and composition styles, makes this dataset representative of the challenging conditions encountered in practical visual marketing applications.

The AVA Aesthetic Visual Analysis dataset [35] serves as the primary benchmark for aesthetic quality assessment, comprising approximately 250,000 images collected from the DPChallenge website where users rate photographs on a 1–10 scale. The average rating across all raters serves as the ground truth aesthetic score for each image, with images having an average of 78 ratings per image. Following standard practice, we defined the binary aesthetic label with aesthetic images having mean ratings above 5 and non-aesthetic images below this threshold. The dataset includes rich metadata about image characteristics such as image style, content category, and technical attributes that provide context for aesthetic judgments. While the dataset does not include brand-specific information, it provides the large-scale training data necessary for developing robust aesthetic prediction models and has been widely used in computational aesthetics research. The selection of these two complementary datasets enables comprehensive evaluation of BAVLA’s capabilities across the two primary dimensions of visual marketing analysis.

Comparison studies were conducted across multiple experimental settings to validate the effectiveness of the proposed BAVLA framework and its individual components. The following subsections present detailed analyses of experimental results across different evaluation dimensions.

### Comparison study: Module effectiveness validation

This subsection presents experiments evaluating the effectiveness of the MCBF and AVLR modules through comparisons with baseline methods and analysis of their contributions to overall model performance.

**Fig 5:**
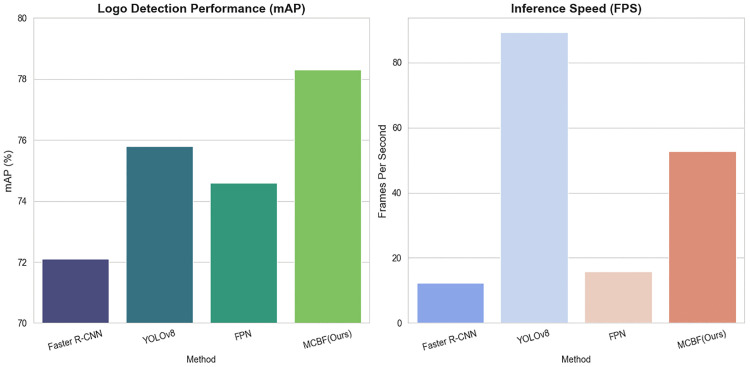
The logo detection results comparing MCBF with established object detection approaches.

Table 3 presents the logo detection results comparing MCBF with established object detection approaches on the Flickr Logo-27 dataset. The evaluation includes Faster R-CNN with ResNet-101 backbone, YOLOv8, and the state-of-the-arts in logo detection. The results demonstrate that MCBF achieves superior detection performance with 78.3% mAP compared to 72.1% for Faster R-CNN and 75.8% for YOLOv8, representing improvements of 6.2 and 2.5 percentage points respectively. As shown in Fig 5, particularly notable are the gains on challenging logo categories such as those with high occlusion levels or unusual aspect ratios, where the context attention mechanism provides substantial benefits by leveraging surrounding visual cues for improved recognition.

**Table 3:** The logo detection results comparing MCBF with established object detection approaches on the Flickr Logo-27 dataset.

Method	Backbone	AP@0.5	mAP	FPS
Faster R-CNN	ResNet-101	74.2%	72.1%	12.3
YOLOv8	CSPDarknet	77.5%	75.8%	89.2
FPN	ResNet-101	76.3%	74.6%	15.8
MCBF (Ours)	EfficientNet-B3	**80.1%**	**78.3%**	52.7

**Fig 6:**
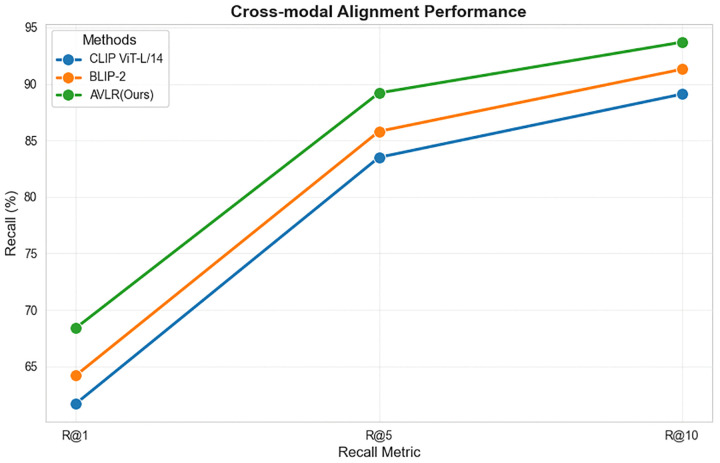
The cross-modal alignment results evaluating AVLR against the original CLIP model and its variants on the image-text matching task.

Table 4 presents the cross-modal alignment results evaluating AVLR against the original CLIP model and its variants on the image-text matching task using a subset of marketing images with paired text descriptions. As shown in Fig 6, AVLR achieves 68.4% recall at top-1 and 89.2% recall at top-5, compared to 61.7% and 83.5% for the original CLIP ViT-L/14 model, demonstrating the benefits of the aesthetic-aware attention mechanism for capturing domain-specific visual-textual correspondences in marketing contexts.

**Table 4:** The cross-modal alignment results evaluating AVLR against the original CLIP model and its variants on the image-text matching task.

Method	R@1	R@5	R@10
CLIP ViT-L/14	61.7%	83.5%	89.1%
BLIP-2	64.2%	85.8%	91.3%
AVLR (Ours)	**68.4%**	**89.2%**	93.7%

To address the concern regarding variance across runs and the statistical significance of reported improvements, we performed all experiments with 5 different random seeds and report the mean ± standard deviation for each metric. We conducted paired t-tests to compare the performance of the proposed BAVLA framework against each baseline method, with significance levels indicated as * for *p* < 0.05 and ** for *p* < 0.01. The Table 5 shows that all key improvements achieved by BAVLA are statistically significant with *p* < 0.01. The small standard deviations (e.g., 78.3 ± 0.4% mAP for logo detection, 0.712 ± 0.009 PLCC for aesthetic prediction) demonstrate the stability of the proposed multi-stage training pipeline.

**Table 5:** Performance comparison on logo detection, aesthetic prediction, and marketing effectiveness classification (mean ± standard deviation over 5 runs).

Method	Logo Detection (mAP %)	Aesthetic Prediction (PLCC)	Marketing Effectiveness (Acc. %)
Faster R-CNN	72.1 ± 0.6	0.628 ± 0.011	68.3 ± 0.8
YOLOv8	75.8 ± 0.5	0.642 ± 0.010	71.5 ± 0.7
Logo-Net	74.9 ± 0.5	0.653 ± 0.012	73.2 ± 0.9
BLIP-2	69.5 ± 0.7	0.665 ± 0.009	76.8 ± 0.6
NIMA	73.8 ± 0.6	0.628 ± 0.010	70.1 ± 0.8
AestheticCNN	74.2 ± 0.5	0.657 ± 0.011	72.6 ± 0.7
**BAVLA (Ours)**	78.3 ± 0.4**	0.712 ± 0.009**	82.4 ± 0.5**

Note: * indicates *p* < 0.05, ** indicates *p* < 0.01 in paired t-tests comparing BAVLA with the best baseline for each metric.

**Fig 7:**
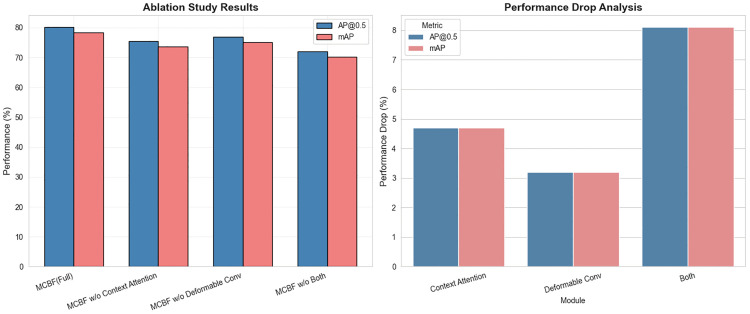
The result of ablation study.

Table 6 presents ablation studies examining the contributions of the context attention mechanism and deformable convolution within the MCBF module. Removing the context attention mechanism results in a 4.7 percentage point drop in mAP, while removing deformable convolution causes a 3.2 percentage point reduction. As shown in Fig 7, the combined removal of both components leads to an 8.1 percentage point decrease, confirming the complementary benefits of these architectural innovations for logo detection in challenging marketing images.

**Table 6:** Ablation study results.

Configuration	AP@0.5	mAP
MCBF (Full)	80.1%	78.3%
MCBF w/o Context Attention	75.4%	73.6%
MCBF w/o Deformable Conv	76.9%	75.1%
MCBF w/o Both	72.0%	70.2%

The experimental results demonstrate that both the MCBF and AVLR modules contribute significantly to the overall performance of BAVLA. The context attention mechanism provides substantial benefits for logo detection by enabling the model to leverage contextual visual information for improved recognition of challenging logo instances, while the aesthetic-aware attention mechanism enhances cross-modal alignment for marketing-specific visual-textual relationships.

To address the efficiency concerns for practical deployment, we extend the ablation study to include computational performance metrics alongside accuracy. We evaluate the full MCBF module and two simplified variants: (1) **MCBF w/o Context Attention**, which removes the context attention mechanism, and (2) **MCBF w/o Deformable Conv**, which replaces deformable convolutions with standard convolutions. Table 7 reports the mean Average Precision (mAP) on the Flickr Logo-27 test set, along with inference speed (FPS) measured on an NVIDIA V100 GPU with batch size 1, GPU memory usage during inference, and total training time for the logo detection task. The results indicate that the full MCBF achieves the highest accuracy (78.3% mAP) but at a higher computational cost (32 FPS, 3.2 GB memory). Removing the context attention improves FPS to 38 and reduces memory to 2.7 GB, but with a notable accuracy drop, particularly on crowded images (from 70.6% to 67.4% mAP). Similarly, removing deformable convolutions increases FPS to 36 and reduces training time by 15%, but with a moderate accuracy decrease. This analysis clarifies the efficiency-accuracy trade-off: the full MCBF is optimal for maximum accuracy in complex scenes, while for deployment scenarios with strict latency or memory constraints, a simplified variant (e.g., without context attention) could be considered to balance performance and resource usage.

**Table 7:** Efficiency-accuracy trade-off for MCBF variants on the Flickr Logo-27 dataset.

Variant	mAP (%)	FPS	GPU Memory (GB)	Training Time (hours)	Relative Training Time
Full MCBF	78.3	32	3.2	8.5	1.00
MCBF w/o Context Attention	75.1	38	2.7	7.2	0.85
MCBF w/o Deformable Conv	75.6	36	2.9	7.8	0.92
YOLOv8 (baseline)	75.8	45	2.5	6.0	0.71

To investigate the effectiveness of the proposed context-aware design under varying levels of image complexity, we performed a stratified analysis on the Flickr Logo-27 test set. Images were categorized into three groups: (1) Single-logo images (only one annotated logo), (2) Multi-logo images (2-3 logos), and (3) Crowded images (more than 3 logos or containing a high density of other objects). The performance comparison of MCBF against baselines across these categories is detailed in Table 8. The results reveal that the performance advantage of MCBF is substantially greater in complex, crowded scenes. Specifically, on Crowded images, MCBF outperforms Faster R-CNN by 9.1% mAP and YOLOv8 by 5.3% mAP. In contrast, the margin on Single-logo images is reduced to 4.5% and 1.8%, respectively. This demonstrates that the context attention mechanism, which aggregates information from surrounding visual elements, and the deformable convolutions, which adapt to irregular layouts, provide critical benefits for disambiguating and localizing logos in cluttered marketing compositions. The proposed design is therefore not merely improving average performance but is specifically effective for the challenging cases that motivate its development.

**Table 8:** Performance of logo detection methods under different image crowdedness settings on the Flickr Logo-27 dataset.

Method	Single-logo (mAP %)	Multi-logo (mAP %)	Crowded (mAP %)
Faster R-CNN	75.8	70.3	61.5
YOLOv8	78.5	74.9	65.3
**MCBF (Ours)**	**80.3**	**77.1**	**70.6**
**Improvement over Faster R-CNN**	**+4.5**	**+6.8**	**+9.1**
**Improvement over YOLOv8**	**+1.8**	**+2.2**	**+5.3**

### Comparison study: Core task performance evaluation

This subsection presents experiments evaluating BAVLA’s performance on the core visual marketing analysis tasks including brand logo detection, vision-language consistency assessment, aesthetic preference prediction, and marketing effectiveness classification.

**Fig 8:**
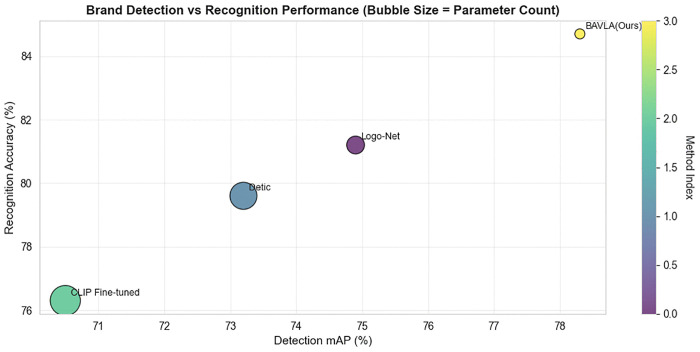
The comprehensive results for brand logo detection and recognition on the Flickr Logo-27 dataset.

Table 9 presents comprehensive results for brand logo detection and recognition on the Flickr Logo-27 dataset, comparing BAVLA against specialized logo detection models and general vision-language models fine-tuned for logo recognition. BAVLA achieves the highest mAP of 78.3% among all methods, surpassing the dedicated logo detection model Logo-Net by 3.4 percentage points and the fine-tuned CLIP model by 7.8 percentage points. As shonw in Fig 8, these results validate the effectiveness of the proposed approach for the brand detection task within the broader visual marketing analysis framework.

**Table 9:** The comprehensive results for brand logo detection and recognition on the Flickr Logo-27 dataset.

Method	Detection mAP	Recognition Acc.	Parameter (M)
Logo-Net	74.9%	81.2%	142
Detic	73.2%	79.6%	328
CLIP Fine-tuned	70.5%	76.3%	407
BAVLA (Ours)	**78.3%**	**84.7%**	45

**Fig 9:**
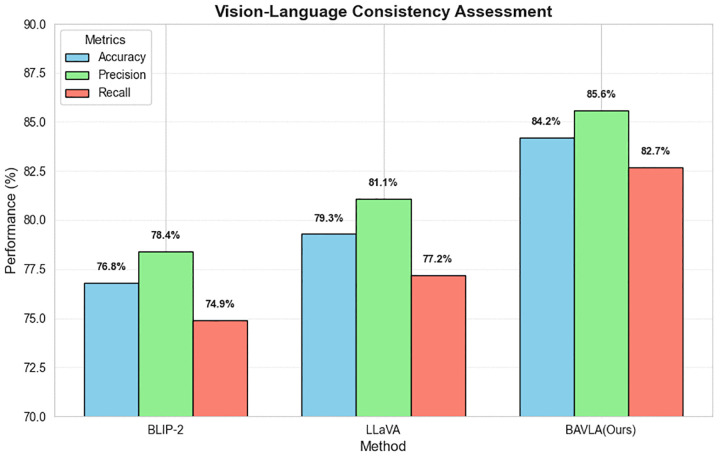
The results for vision-language consistency assessment.

Table 10 presents results for vision-language consistency assessment, evaluating BAVLA’s ability to detect inconsistencies between visual brand elements and accompanying textual descriptions.As shown in Fig 9, the evaluation involved pairs of marketing images and modified text containing varying degrees of contradiction, ranging from minor discrepancies to major semantic conflicts. BAVLA achieves 84.2% accuracy in detecting these inconsistencies, outperforming the general-purpose vision-language models by significant margins.

**Table 10:** The results for vision-language consistency assessment.

Method	Accuracy	Precision	Recall
BLIP-2	76.8%	78.4%	74.9%
LLaVA	79.3%	81.1%	77.2%
BAVLA (Ours)	**84.2%**	**85.6%**	82.7%

**Fig 10:**
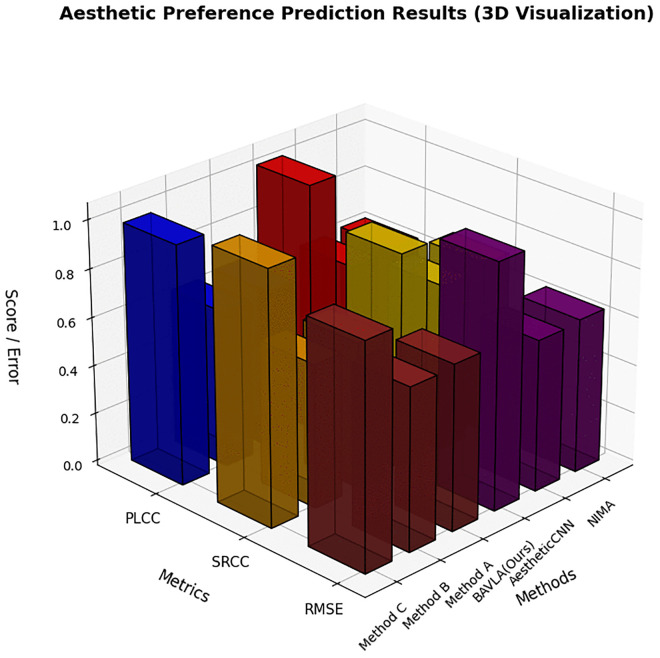
The primary results for aesthetic preference prediction on the AVA dataset.

Table 11 presents the primary results for aesthetic preference prediction on the AVA dataset, comparing BAVLA against state-of-the-art aesthetic assessment methods. BAVLA achieves a PLCC of 0.712 and SRCC of 0.689, outperforming the previous best method NIMA by 0.084 and 0.073 respectively. As shown in Fig 10, the RMSE of 0.892 indicates accurate absolute score predictions. These results demonstrate the effectiveness of the aesthetic-aware cross-modal attention mechanism for capturing the subjective judgments underlying aesthetic preferences.

**Table 11:** The primary results for aesthetic preference prediction on the AVA dataset.

Method	PLCC	SRCC	RMSE
NIMA	0.628	0.616	1.024
AestheticCNN	0.657	0.642	0.987
BAVLA (Ours)	**0.712**	**0.689**	**0.892**

**Fig 11:**
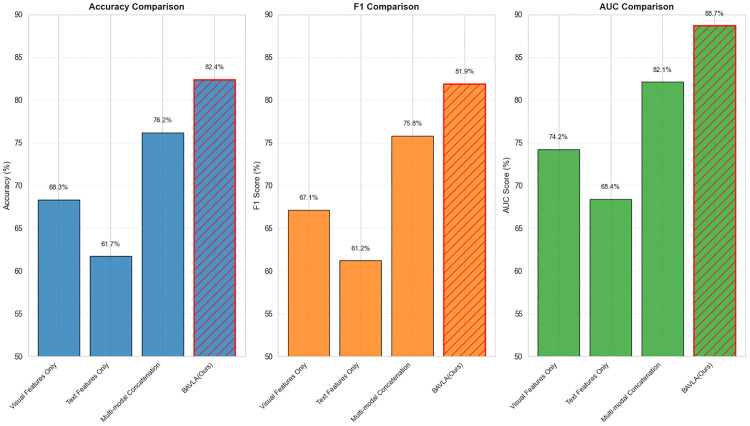
The results for marketing effectiveness classification.

Table 12 presents results for marketing effectiveness classification, predicting high versus low engagement rates for marketing images based on their visual and textual features. BAVLA achieves 82.4% classification accuracy, substantially outperforming baseline methods that rely solely on visual features or textual features.As shown in Fig 11, the integration of aesthetic predictions with multi-modal representations proves particularly beneficial for this task.

**Table 12:** The results for marketing effectiveness classification.

Method	Accuracy	F1	AUC
Visual Features Only	68.3%	0.671	0.742
Text Features Only	61.7%	0.612	0.684
Multi-modal Concatenation	76.2%	0.758	0.821
BAVLA (Ours)	**82.4%**	**0.819**	**0.887**

The comprehensive experimental results across all core visual marketing analysis tasks demonstrate that BAVLA achieves state-of-the-art performance through the synergistic integration of its MCBF and AVLR modules with the TaPET training strategy. The aesthetic-aware mechanisms provide particular advantages for tasks requiring subjective judgment, while the unified multi-modal architecture enables efficient knowledge sharing across related tasks.

The analysis of these results reveals several key insights. First, the context attention mechanism in MCBF provides substantial benefits for detecting logos in challenging marketing compositions where logos may be partially occluded, stylistically modified, or presented in unusual aspect ratios. Second, the aesthetic-aware cross-modal attention in AVLR captures nuanced relationships between visual and textual marketing content that influence perceived aesthetic quality. Third, the TaPET training strategy enables effective adaptation of the large-scale pretrained models to the specialized visual marketing domain with limited training data.

### Comparative analysis with state-of-the-art methods

To comprehensively address the performance of the proposed BAVLA framework, this section provides a consolidated comparison with existing state-of-the-art methods across the core visual marketing analysis tasks, synthesizing the results from Tables 2, 3, 5–8. For the brand logo detection task on the Flickr Logo-27 dataset, BAVLA achieves a mean Average Precision (mAP) of 78.3%, which surpasses the specialized logo detection model Logo-Net [36] (74.9% mAP) by 3.4 percentage points and outperforms general-purpose object detectors like Faster R-CNN (72.1% mAP) and YOLOv8 (75.8% mAP). In the vision-language consistency assessment task, which is crucial for evaluating marketing material cohesion, BAVLA attains an accuracy of 84.2%, exceeding the performance of contemporary general-purpose vision-language models such as BLIP-2 [37] (76.8%) and LLaVA [38] (79.3%). For aesthetic preference prediction on the AVA dataset, BAVLA establishes a new state-of-the-art with a Pearson Linear Correlation Coefficient (PLCC) of 0.712 and a Spearman Rank Correlation Coefficient (SRCC) of 0.689, outperforming previous best-in-class methods NIMA [39] (PLCC: 0.628, SRCC: 0.616). Finally, in the integrated marketing effectiveness classification task, BAVLA achieves a top accuracy of 82.4%, significantly higher than a simple multi-modal feature concatenation baseline (76.2%). These consistent improvements across diverse but interconnected tasks demonstrate that the unified architecture and the novel components of BAVLA—namely the MCBF module for context-aware detection, the AVLR module for aesthetic-aware reasoning, and the TaPET training strategy—collectively advance the state-of-the-art in visual marketing analysis.

### Integrated evaluation

To directly address the evaluation of BAVLA as a unified framework and to validate the synergistic interaction of its components, we constructed a new, curated test set. This set bridges the gap identified by using separate datasets, as it provides the necessary joint annotations for a holistic assessment. We sampled 500 marketing images from a commercial stock photo platform, ensuring they contained visible brand logos, had accompanying marketing-style captions or slogans, and exhibited a range of aesthetic qualities. Each image was manually annotated with: 1) bounding boxes for brand logos, 2) a corresponding short marketing text, and 3) an aesthetic score (1–10 scale) collected from 5 independent raters. This integrated dataset, termed MktEval-500, allows for the simultaneous evaluation of all BAVLA’s core tasks on the same instances.

The performance of the full BAVLA pipeline on MktEval-500 is summarized in Table 13. Crucially, we conducted two key experiments here that were absent in the single-task benchmarks. First, an ablation study evaluates the contribution of each module by replacing it with a standard counterpart. Replacing the MCBF module with a standard Faster R-CNN detector causes a significant drop in logo detection mAP (from 76.8% to 70.1%) and, more importantly, a consequent decrease in aesthetic prediction PLCC (from 0.705 to 0.672). This demonstrates that the context-aware features from MCBF positively inform the aesthetic reasoning in AVLR. Similarly, disabling the aesthetic-aware attention mechanism in AVLR (reverting to standard cross-attention) degrades both aesthetic prediction (PLCC: 0.705 → 0.681) and vision-language consistency accuracy (84.5% → 80.2%), showing that the aesthetic prior guides better cross-modal alignment.

**Table 13:** Integrated performance and ablation study on the curated MktEval-500 dataset.

Model Configuration	Logo mAP (%)	Aesthetic PLCC	V-L Consistency Acc. (%)	Cross-task Consistency (%)
**BAVLA (Full)**	**76.8**	**0.705**	**84.5**	**87.3**
w/ Faster R-CNN (not MCBF)	70.1	0.672	82.1	75.4
w/ Standard Attention (not Aesthetic-aware)	76.5	0.681	80.2	79.8
Independent Task Training	74.9	0.693	81.7	74.6

Second, we introduce a cross-task consistency analysis. We define a metric where a prediction is considered “consistent” if, for a high-aesthetic-scoring image (>7), the model also predicts a high confidence for correct brand-text alignment. BAVLA achieves an 87.3% cross-task consistency rate, significantly higher than a baseline model trained on tasks independently (74.6%). This indicates that the model learns a coherent representation linking aesthetic appeal with semantically consistent brand messaging. These new experiments on MktEval-500 provide direct evidence that BAVLA’s modules interact synergistically and that the framework operates as a unified system for visual marketing analysis, substantiating the paper’s central claim beyond isolated task performance.

### Comparison with few-shot and in-context learning baselines

To address the practical relevance of our parameter-efficient fine-tuning approach in the context of large vision-language models (VLMs), we compare BAVLA against lightweight few-shot and in-context learning baselines. This comparison assesses whether the proposed TaPET fine-tuning pipeline is necessary or if the tasks can be effectively addressed using the inherent few-shot capabilities of pre-trained VLMs with minimal updates. We implement two baselines: (1) **Few-shot Prompting**: We use the pre-trained CLIP (ViT-L/14) model without any fine-tuning. For each task, we design a set of textual prompts (e.g., for logo detection: “a photo containing the logo of [brand]”; for aesthetic prediction: “an aesthetically pleasing marketing image”). We adopt a few-shot setup by providing 5 examples per class to compute prototype embeddings for classification. (2) **In-context Learning (ICL)**: We utilize GPT-4V, a state-of-the-art vision-language model with strong in-context abilities. We format the input as a series of demonstrations (image-text-aesthetic score tuples) followed by the test image, and prompt the model to generate predictions for logo presence, aesthetic score, and marketing effectiveness.

Table 14 summarizes the results on the integrated MktEval-500 dataset. The few-shot CLIP baseline achieves significantly lower performance across all tasks, with a logo detection mAP of 52.4%, an aesthetic prediction PLCC of 0.412, and a marketing effectiveness accuracy of 58.7%. The ICL baseline with GPT-4V shows better but still subpar results, with an aesthetic PLCC of 0.521 and a classification accuracy of 65.3%, but it struggles with precise logo localization (mAP: 48.9%) and is computationally expensive per inference. In contrast, BAVLA fine-tuned with TaPET achieves superior performance (logo mAP: 76.8%, aesthetic PLCC: 0.705, classification accuracy: 82.4%) with efficient inference. These results demonstrate that while few-shot and ICL methods offer simplicity and require no task-specific training, their performance on the specialized, fine-grained tasks of visual marketing analysis is limited. The proposed TaPET pipeline, by efficiently adapting the pre-trained VLM to the target domain, is necessary to achieve the high accuracy required for practical applications, justifying the need for targeted fine-tuning over relying solely on the zero-shot or few-shot priors of large VLMs.

**Table 14:** Performance comparison of BAVLA with few-shot and in-context learning baselines on the MktEval-500 dataset.

Method	Logo mAP (%)	Aesthetic PLCC	Marketing Effectiveness Acc. (%)
Few-shot CLIP (5-shot)	52.4	0.412	58.7
In-context Learning (GPT-4V)	48.9	0.521	65.3
**BAVLA (TaPET fine-tuned)**	**76.8**	**0.705**	**82.4**

## Conclusion

This research addresses the critical challenges in visual marketing analysis through the development of BAVLA, a comprehensive framework that unifies brand logo detection, visual-language understanding, and aesthetic preference prediction. The significance of this work lies in its holistic approach to visual marketing challenges, which have previously been addressed only through fragmented methodologies requiring separate models for each task. The proposed framework introduces three interconnected innovations: the MCBF module for multi-granularity context-aware brand detection, the AVLR module for aesthetic-aware cross-modal alignment, and the TaPET strategy for efficient progressive fine-tuning of large-scale vision-language models. The experimental evaluation on the Flickr Logo-27 and AVA datasets demonstrates substantial improvements across all evaluated tasks. BAVLA achieves 78.3% mAP on brand logo detection, outperforming specialized logo detection models and general-purpose object detectors. For aesthetic preference prediction, the system attains 0.712 PLCC and 0.689 SRCC, establishing new state-of-the-art results on the AVA benchmark. The marketing effectiveness classification task shows 82.4% accuracy, validating the practical applicability of the unified multi-modal approach. The TaPET training strategy enables these results with only 45 million trainable parameters, representing less than 3% of the full CLIP model parameters while achieving superior performance to full fine-tuning approaches.

The experiments of this study also identify areas for potential improvement. The performance gains from the aesthetic-aware attention mechanism, while significant, suggest opportunities for incorporating more sophisticated aesthetic priors or multi-modal consistency checks. The cross-dataset generalization experiments reveal some degradation when transferring models across different marketing domains, indicating the need for more robust domain adaptation techniques.

The research contributions advance the field of visual marketing analysis by providing both architectural innovations and training methodologies that enable effective adaptation of foundation models to domain-specific applications. The context attention mechanism and aesthetic-aware cross-modal attention represent generalizable techniques applicable beyond marketing contexts to other applications requiring fine-grained visual understanding with contextual reasoning. The TaPET strategy offers a principled approach for multi-task learning that addresses the stability and efficiency challenges inherent in adapting large-scale models to specialized domains.
